# Reduced Inter-Voxel White Matter Integrity in Subjective Cognitive Decline: Diffusion Tensor Imaging With Tract-Based Spatial Statistics Analysis

**DOI:** 10.3389/fnagi.2022.810998

**Published:** 2022-02-23

**Authors:** Yi-Ping Chao, Po-Ting Bertram Liu, Pei-Ning Wang, Chia-Hsiung Cheng

**Affiliations:** ^1^Department of Computer Science and Information Engineering, Chang Gung University, Taoyuan City, Taiwan; ^2^Department of Neurology, Chang Gung Memorial Hospital, Taoyuan City, Taiwan; ^3^Department of Occupational Therapy and Graduate Institute of Behavioral Sciences, Chang Gung University, Taoyuan City, Taiwan; ^4^Laboratory of Brain Imaging and Neural Dynamics (BIND Lab), Chang Gung University, Taoyuan City, Taiwan; ^5^Division of General Neurology, Department of Neurological Institute, Taipei Veterans General Hospital, Taipei City, Taiwan; ^6^Department of Neurology, National Yang Ming Chiao Tung University, Taipei City, Taiwan; ^7^Healthy Aging Research Center, Chang Gung University, Taoyuan City, Taiwan; ^8^Department of Psychiatry, Chang Gung Memorial Hospital, Taoyuan City, Taiwan

**Keywords:** subjective memory complaint (SMC), diffusion tensor imaging (DTI), white matter, local diffusion homogeneity (LDH), Alzheimer’s disease (AD)

## Abstract

Subjective cognitive decline (SCD), a self-reported worsening in cognition concurrent with normal performance on standardized neuropsychological tests, has gained much attention due to its high risks in the development of mild cognitive impairments or Alzheimer’s disease. The existing cross-sectional diffusion tensor imaging (DTI) studies in SCD have shown extremely controversial findings. Furthermore, all of these studies investigated diffusion properties within the voxel, such as fractional anisotropy, mean diffusivity, or axial diffusivity (DA). However, it remains unclear whether individuals with SCD demonstrate alterations of diffusion profile between voxels and their neighbors, as indexed by local diffusion homogeneity (LDH). We selected 30 healthy controls (HCs) and 23 SCD subjects to acquire their whole-brain DTI. Diffusion images were compared using the tract-based spatial statistics method. Diffusion indices with significant between-group tract clusters were extracted from each individual for further region-of-interest (ROI)-based comparisons. Our results showed that subjects with SCD demonstrated reduced LDH in the left superior frontal gyrus (SFG) and DA in the right anterior cingulate cortex compared with the HC group. In contrast, the SCD group showed higher LDH values in the left lingual gyrus (LG) compared with the HC group. Notably, LDH in the left SFG was significantly and negatively correlated with LDH in the left LG. In conclusion, white matter (WM) integrity in the left SFG, right ACC, and left LG is altered in SCD, suggesting that individuals with SCD exhibit detectable changes in WM tracts before they demonstrate objective cognitive deficits.

## Introduction

Although there is a consensus that amnestic mild cognitive impairment (aMCI) stands in an intermediate stage between normal aging and Alzheimer’s disease (AD), several lines of evidence from large-cohort follow-up studies have suggested subjective cognitive decline (SCD) as a pre-MCI stage in the AD spectrum (Jessen et al., 2010; Albert et al., 2011; Molinuevo et al., 2017). SCD is defined as a self-reported cognitive decline, particularly relevant for the memory domain, concurrent with normal performance (adjusted by age and education) in the assessments of objective neuropsychological functions; furthermore, the identification of SCD cannot be explained by other neurological diseases (e.g., MCI and AD), psychiatric diseases (e.g., anxiety disorder and depressive disorder), medication, or substance use (Jessen et al., 2014, 2020). Longitudinal studies have demonstrated that compared with older adults without SCD, those with SCD showed a greater risk for conversion to AD or aMCI (Koppara et al., 2015; Buckley et al., 2016; Slot et al., 2019; Mazzeo et al., 2020). A recent meta-analytic study has further indicated that approximately 25% of older adults with SCD would develop MCI in the next 4 years (Mitchell et al., 2014). Therefore, accurate identification of SCD is clinically important for better targeted early intervention and for monitoring disease progression. Since neuropsychological assessments are not sensitive enough to distinguish individuals with SCD from those without SCD, neuroimaging technology provides a promising window to study the neural basis of SCD.

Reduced gray matter (GM) volume/density of the hippocampus (Saykin et al., 2006; Perrotin et al., 2015), entorhinal cortex (Jessen et al., 2006; Ryu et al., 2017), or frontal regions (Hong et al., 2015) has been reported in SCD as compared with normal controls without SCD. However, several lines of evidence suggest that loss of axonal integrity is independent of, and occurs earlier than, corresponding GM atrophy (Salat et al., 2010; Selnes et al., 2012). At present, there are several studies using diffusion tensor imaging (DTI) to discern microstructural changes between those with and without SCD. However, the results are extremely inconsistent. Most of the studies have shown white matter (WM) disturbances in SCD as compared with healthy controls (HCs), but the deteriorated regions exhibited great variabilities among these studies (Salat et al., 2010; Selnes et al., 2012; Hong et al., 2015; Li et al., 2016; Brueggen et al., 2019; Luo et al., 2019; Shao et al., 2019). There were still some studies reporting no significant changes in WM integrity between those with and without SCD (Wang et al., 2012; Kiuchi et al., 2014; Wen et al., 2019). One of the major reasons leading to these controversial data was potentially due to the analysis strategy. The majority of DTI studies applied region-of-interest (ROI)-level analysis, while very few studies used tract-based spatial statistics (TBSS), which is a method for voxel-based analysis of WM diffusion data to improve detection and localization of WM changes across individuals and groups (Smith et al., 2006). There have been four DTI studies with TBSS analysis in SCD research (Selnes et al., 2012; Wang et al., 2012; Li et al., 2016; Brueggen et al., 2019). Selnes et al. (2012) found that compared with normal controls, individuals with SCD showed higher mean diffusivity (MD) and radial diffusivity (DR) in the WM tracts underlying the posterior cingulate, retrosplenial, and middle temporal cortices. Higher values of MD in SCD were also reported in the other two studies but with a widespread area, including the splenium of the corpus callosum, internal capsule, external capsule, and superior and inferior longitudinal fasciculus (Li et al., 2016; Brueggen et al., 2019). Increased MD and DR have been associated with myelin damage or cell membrane deterioration (Song et al., 2002; Pievani et al., 2010; Bozzali et al., 2012). In contrast, Wang et al. (2012) did not find any significant WM changes in fractional anisotropy (FA), MD, or DR in the comparisons between those with and without SCD. Thus, the exact pathological changes of cortical microstructures in SCD remain unclear (Sun et al., 2015).

The vast majority of DTI studies derived parameters reflecting diffusion properties within the voxel, such as FA or MD. Notably, a previous study has demonstrated that local diffusion homogeneity (LDH) could measure the local coherence of water molecule diffusion profile between voxels and their neighbors (Gong, 2013). This metric is considered to be complementary to the traditional diffusion indices (i.e., FA and MD) and provides additional information into the WM changes. The abnormalities in LDH have been increasingly reported in patients with schizophrenia (Zhuo et al., 2016), stroke (Liu et al., 2017), amyotrophic lateral sclerosis (Du et al., 2019), and vascular cognitive impairment without dementia (Chen et al., 2018). However, to the best of our knowledge, this novel DTI index (i.e., LDH) has never been used for evaluating microstructural alterations of WM in SCD.

In summary, this study aimed to determine whether individuals with SCD showed WM changes using DTI and TBSS analysis, which minimizes registration errors and personal evaluation bias as compared with ROI analysis. In addition to conventional intra-voxel diffusion measurements, inter-voxel diffusion metrics (i.e., LDH) were also applied to examine microstructural changes of WM in SCD. Finally, we also examined whether changes in WM integrity were correlated with the performance of neuropsychological tests.

## Materials and Methods

### Participants

Initially, 30 HCs and 28 individuals with SCD were selected between 2016 and 2018. Inclusion criteria for both groups were (1) age of 50 years or above; (2) no major neurological and psychiatric disorders that potentially have influences on cognitive functions; (3) no hearing impairment and normal or corrected-to-normal vision; and (4) normal cognitive performance as evaluated by neuropsychological assessments (Table 1). Subjects with SCD were referred from the memory clinic of Taipei Veterans General hospital due to their worries about self-perceived consistent cognitive decline. Furthermore, they reported a complaint on at least one of the specific cognitive tasks compared with the ability two years earlier, as evaluated by a 12-item questionnaire (Cheng et al., 2020, 2021). Importantly, their subjective complaints of cognitive deterioration were confirmed by family members or close friends (Buckley et al., 2016). According to the research criteria proposed by the Subjective Cognitive Decline Initiative (SCD-I) Working Group, depressive symptoms that reach a threshold of clinical disorder should be considered as exclusion criteria (Jessen et al., 2014, 2020). In this study, we excluded three subjects from the SCD group as their Geriatric Depression Scale (GDS) scores were > 5 (Yesavage et al., 1982; Almeida and Almeida, 1999). In addition, one subject with chronic headache and one subject with Meniere’s disease were excluded. Therefore, the data from a total of 30 HCs (mean age: 67.47 ± 7.96 years, 11 men) and 23 SCD (mean age: 67.09 ± 9.00 years, 7 men) were finally analyzed (Table 1).

**TABLE 1:** Demographic variables and neuropsychological measures as mean ± SD.

	HC (*n* = 30)	SCD (*n* = 23)	*P*-values
**Demographic data**			
Sex (male/female)	11/19	7/16	0.643
Age (years)	67.47 ± 7.96	67.09 ± 9.00	0.871
Education (years)	13.8 ± 2.33	12.48 ± 3.49	0.104
**Neuropsychological data**			
GDS	0.97 ± 1.45	2.17 ± 1.64	**0.008**
MMSE	29.03 ± 1.00	29.17 ± 1.07	0.625
*CVVLT*			
Total	31.47 ± 3.37	31.09 ± 4.10	0.713
Delayed	8.40 ± 0.93	8.39 ± 0.99	0.974
*Logical memory A*			
Immediate	15.20 ± 3.62	16.09 ± 3.48	0.373
Delayed	14.47 ± 4.03	14.39 ± 3.37	0.943
*CFT*			
Copy	32.70 ± 2.48	32.70 ± 2.16	0.995
Immediate	24.63 ± 6.22	24.07 ± 6.60	0.750
Delayed	24.13 ± 6.52	23.24 ± 6.52	0.623
Verbal fluency test	18.77 ± 4.30	18.87 ± 5.56	0.940
*BNT*			
Spontaneous	26.63 ± 2.44	27.74 ± 1.89	0.078
Semantic cues	0.50 ± 0.73	0.43 ± 0.79	0.757
Phonemic cues	1.83 ± 1.39	0.87 ± 0.81	**0.005**
*Digit span test*			
Forward	8.4 ± 1.10	8.35 ± 0.71	0.844
Backward	5.6 ± 1.57	5.70 ± 1.40	0.818
*Trail making test*			
Part A (s)	18.33 ± 16.23	14.00 ± 11.39	0.281
Part B (s)	38.13 ± 26.18	31.74 ± 14.60	0.298

*SD, standard deviation; HC, healthy control; SCD, subjective cognitive decline; GDS, Geriatric Depression Scale; MMSE, Mini-Mental State Examination; CVVLT, Chinese Version Verbal Learning Test; Logical memory A, Logical memory A of Wechsler Memory Scale; CFT, Rey-Osterrieth Complex Figure Test; BNT, Boston Naming Test. P values with significance are highlighted in bold fonts.*

This study was approved by the Institutional Review Board of Taipei Veterans General Hospital (approval code: 2016-06-001B) and was performed in accordance with approved guidelines and regulations. Written informed consent was obtained from all the participants after detailed descriptions of experimental procedures.

### Diffusion Tensor Image Acquisition

Magnetic resonance imaging (MRI) was performed using a 3T MRI system (Discovery MR750, Wisconsin, United States) with an 8-channel head coil at Taipei Veterans General Hospital, Taipei, Taiwan. DTI data were acquired using the following setting: time repetitive (TR)/time echo (TE) = 10,000/80.8 ms, number of average = 3, *b*-value = 1,000 s/mm^2^, slice thickness = 2 mm, number of diffusion sampling directions = 30, number of null images = 3 (*b*-value = 0), field of view (FOV) = 256 mm, resolution = 256 × 256, and number of axial slices to cover whole brain = 78. The anatomical T1-weighted image was acquired using the following setting: 3D fast spoiled gradient echo (FSPGR) sequence, TR/TE/time inversion (TI) = 9.384/4.036/450 ms, flip angle = 12°, FOV = 256 mm, resolution = 256 × 256, slice thickness = 1 mm, and number of axial slice = 172.

### Diffusion Tensor Imaging Analysis

DTI data were analyzed using a MATLAB toolbox, PANDA (Pipeline for Analyzing braiN Diffusion imAges), which was conducted through FMRIB Software Library (FSL 5.0.9, University of Oxford, United Kingdom).^1^ Based on the pipeline provided by PANDA, first, eddy current distortions and motion artifacts were corrected using the FSL’s eddy correction tool, and then the corrected DTI images were stripped to remove non-brain tissues using the FSL Brain Extraction Tool (Smith, 2002). Second, diffusion index maps including FA, MD, DR, axial diffusivity (DA), and LDH were calculated using the FSL diffusion tensor analysis toolkit (FDT).

Next, TBSS was performed within FSL following the standard pipeline.^2^ All of the FA images from each subject were registered to the Montreal Neurological Institute (MNI) 152 standard space from native space by non-linear transformation through the FSL registration tool, namely, FNIRT. A group-averaged FA skeleton of all subjects was created by thresholding FA > 0.2. Finally, all participants’ FA images were projected onto this skeleton to create normalized skeletonized FA images. Similarly, other diffusion index maps including MD, DA, DR, and LDH images were all registered to the MNI152 standard space using the non-linear transformation of FA images, and individual skeletonized images were generated for voxel-wise statistics between two groups.

**FIGURE 1:**
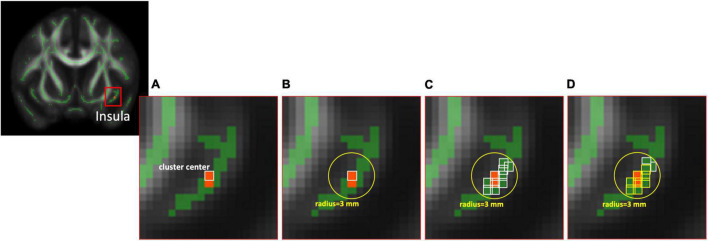
The diagram of ROI selection. **(A)** The cluster center was derived from the statistical analysis between two groups based on voxel-wise TBSS with significant differences. **(B)** The spherical ROIs with radius = 3 mm were estimated using the MarsBaR toolbox. **(C)** The voxels (white bounding) within the FA skeleton based on TBSS were reserved first, then **(D)** the voxels (yellow bounding), which are the same anatomical region of AAL as the cluster center, were selected at last for further ROI-based analysis. ROI, region of interest; TBSS, tract-based spatial statistics; FA, fractional anisotropy; AAL, automated anatomical labeling.

Based on the results of TBSS, locations with significant between-group differences (described in the “Statistics” section) were further analyzed using an ROI-based approach to highlight the structural changes between these two groups. We used the MarsBaR (MARSeille Boîte À Région d’Intérêt), which is a toolbox for statistical parametric mapping (SPM), to estimate the spherical ROIs (radius = 3 and 5 mm, respectively) using the MNI coordinate of cluster center with a significant difference from above voxel-wise statistics between two groups. Finally, the averaged values of diffusion index maps were extracted from the intersection of the estimated spherical ROIs, the anatomical region based on the automated anatomical labeling (AAL) template of the MNI coordinate of the cluster center, and the skeleton derived from the TBSS for further ROI-based statistical analyses (Figure 1).

### Statistics

All data are presented as mean ± standard deviation (SD). To realize the differences of WM structure, voxel-wise comparisons of diffusion index maps within the normalized WM skeleton in MNI152 standard space were performed using the general linear model (GLM) and independent *t*-tests with covariables of age, gender, GDS score, and years of education. The statistical results of group analyses were evaluated using the threshold-free cluster enhancement (TFCE) method (version 174)^3^ in this study. The TFCE method is a non-parametric permutation-based approach that requires no arbitrary definition of voxel-wise or cluster thresholds on neuroimaging data (Smith and Nichols, 2009). The default parameters of 5,000 permutations, *E* = 1 and *H* = 2, were used for TBSS data in each TFCE-based analysis. Clusters were considered significant if they passed a cluster-level threshold of uncorrected *p* < 0.001 with an additional threshold of cluster size 4.

For ROI-based analyses, independent *t*-tests (two-tailed) were performed using Statistical Package for the Social Sciences (SPSS) version 19 (IBM Corporation, New York, United States), and the significance level was set to *p* < 0.05. The effect sizes (Cohen’s *d*) for each comparison were also calculated. The Cohen’s *d* effect sizes between 0.2 and 0.5 were considered small, those between 0.5 and 0.8 were considered moderate, and those over 0.8 were considered large (Cohen, 1992).

## Results

### Subject Characteristics

The two groups did not significantly differ regarding age, gender distribution, and levels of education. Compared with HCs, individuals with SCD demonstrated more depressive symptoms (*p* = 0.008) though they did not fulfill the diagnostic criteria for clinical depression. Although between-group differences were not significant in most of the neuropsychological tests, SCD subjects showed better performance in the phonemic cues of the Boston Naming Test as compared with HCs (*p* = 0.005).

### Whole-Brain Diffusion Tensor Imaging

**FIGURE 2:**
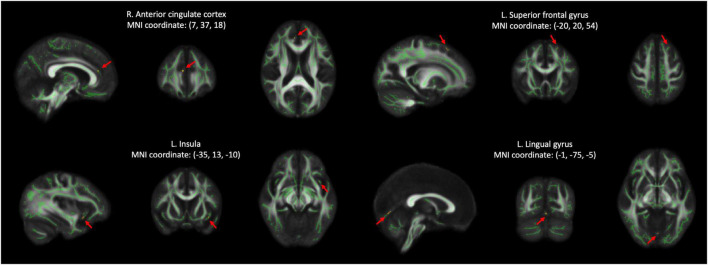
Results of voxel-wise group comparison and TBSS using the TFCE with uncorrected *p* < 0.001 and cluster size ≥ 4. TBSS, tract-based spatial statistics; TFCE, threshold-free cluster enhancement; L, left; R, right; MNI, Montreal Neurological Institute.

The voxel-wise TBSS analysis showed that compared with HCs, SCD had significantly lower DA values of the right anterior cingulate cortex (ACC, cluster size = 4) and lower LDH values of left superior frontal gyrus (SFG, cluster size = 6). In contrast, SCD demonstrated higher values of DA in the left insula (cluster size = 5) and LDH in the left lingual gyrus (LG, cluster size = 4). The detailed results are listed in Figure 2 and Table 2.

**TABLE 2:** Voxel-based group comparisons between HC and SCD (*p* < 0.001 after TFCE, uncorrected, two-tailed, *k* ≧ 2).

Metrics	Location	Hemisphere	*k*	MNI coordinate		
**HC > SCD**						
DA	**Anterior cingulate cortex**	**R**	**4**	7	37	18
			3	8	41	9
DR	Inferior parietal lobule	R	2	43	−38	44
LDH	Insula	R	3	40	−12	1
	Medial frontal gyrus	R	2	5	−15	61
	**Superior frontal gyrus**	**L**	**6**	−20	20	54
**HC < SCD**						
MD	Superior temporal gyrus	R	2	31	3	−17
DA	**Insula**	**L**	**5**	−35	13	−10
	Thalamus	L	2	−21	−27	8
DR	Superior temporal gyrus	R	3	31	3	−17
LDH	**Lingual gyrus**	**L**	**4**	−1	−75	−5

*HC, healthy control; SCD, subjective cognitive decline; TFCE, threshold-free cluster enhancement; MD, mean diffusivity; DA, axial diffusivity; DR, radial diffusivity; LDH, local diffusion homogeneity; k = cluster size; MNI coordinate = cluster center. The regions with k ≧ 4, which are highlighted in bold fonts, are subject to further region-of-interest-based analysis.*

### Regional Analysis of Diffusion Tensor Imaging

**FIGURE 3:**
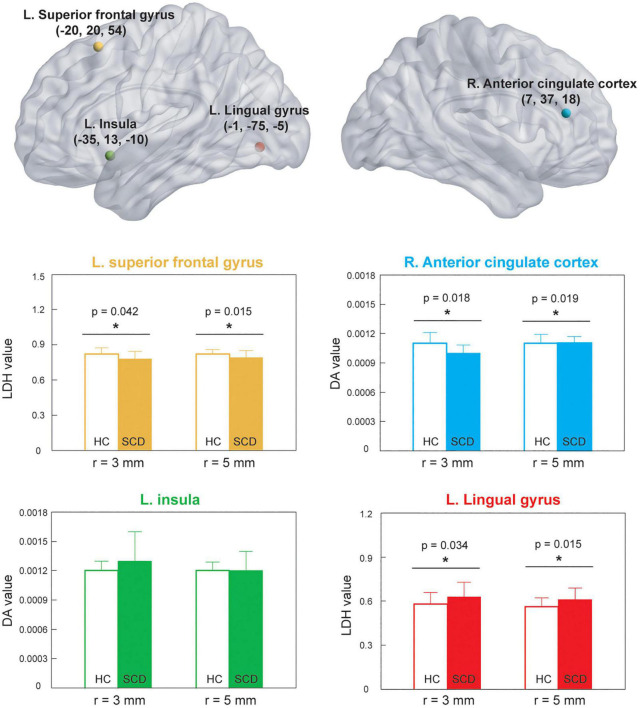
ROI-based analysis was based on the significant locations shown in Figure 2. *p < 0.05. HC, healthy control; SCD, subjective cognitive decline; ROI, region of interest; LDH, local diffusion homogeneity; DA, axial diffusivity; L, left; R, right.

Since the cluster size with significant differences after TFCE were relatively small in this study, we performed additional ROI-wise analysis based on the selected regions with cluster size ≥ 4, including right ACC, left SFG, left insula, and left LG for further validation. Two different sizes of spherical ROI were identified with radius = 3 and 5 mm, respectively, and compared between HCs and SCD (Figure 3). The statistical results revealed that compared with HCs, individuals with SCD demonstrated reduced DA values of the right ACC both in the ROIs with radius = 3 mm (number of voxels = 14, *p* = 0.018, Cohen’s *d* = 0.691) and radius = 5 mm (number of voxels = 24, *p* = 0.019, Cohen’s *d* = 0.610) and reduced LDH values of the left SFG both in the ROIs with radius = 3 mm (number of voxels = 32, *p* = 0.042, Cohen’s *d* = 0.581) and radius = 5 mm (number of voxels = 65, *p* = 0.015, Cohen’s *d* = 0.713). However, individuals with SCD showed higher LDH values of the left LG as compared with the HC group both in the ROIs with radius = 3 mm (number of voxels = 7, *p* = 0.034, Cohen’s *d* = −0.604) and radius = 5 mm (number of voxels = 23, *p* = 0.015, Cohen’s *d* = −0.701). No significant between-group differences of DA values in the left insula were found either in the ROIs with radius = 3 mm (number of voxels = 40, *p* = 0.183, Cohen’s *d* = −0.468) or radius = 5 mm (number of voxels = 86, *p* = 0.191, Cohen’s *d* = −0.390).

### Correlational Analysis

**FIGURE 4:**
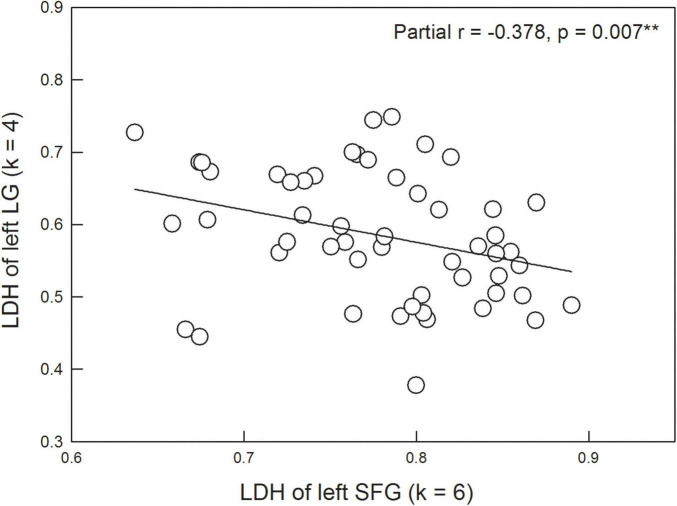
LDH of the left superior frontal gyrus (SFG) is significantly correlated with LDH of the left lingual gyrus (LG) after controlling the age, gender, years of education, and scores of the Geriatric Depression Scale.

Based on the ROIs with significant between-group differences, we further examined the partial correlation coefficient between diffusion metrics and neuropsychological assessments (controlled variables = age, gender, years of education, and GDS scores). No significant results were found after corrections for multiple comparisons. However, it was interesting to note that among the diffusion metrics, LDH values of the left SFG were significantly and negatively correlated with LDH values of the left LG (partial *r* = −0.378, *p* = 0.007) across the HC and SCD groups (Figure 4).

## Discussion

The major aim of this study was to investigate the effects of SCD, a self-perception of subtle cognitive decline with normal cognitive performance evaluated by objective neuropsychological assessments, on the integrity of WM structures using DTI together with TBSS methods. Considering the whole-brain DTI analysis and ROI-based analysis, our results showed that individuals with SCD demonstrated reduced LDH in the left SFG and DA in the right ACC as compared with the HC group. Besides, the SCD group showed higher LDH values in the left LG as compared with the HC group. It was also interesting to note that LDH of left SFG was significantly and negatively correlated with LDH of left LG.

The SFG has been reported to be involved in higher cognitive functions including working memory and episodic memory (Nissim et al., 2016; Alagapan et al., 2019). For example, a previous lesion study showed that compared with HCs, patients with left SFG lesions, particularly in Brodmann area 8, demonstrated impairments in working memory (du Boisgueheneuc et al., 2006). It was also interesting to note that in this lesion study, two patient groups with prefrontal lesions sparing the SFG and the right parietal cortex did not show such deficits, suggesting the particular role of the left SFG in working memory function. Another study with resting-state functional MRI further revealed that compared with HCs, individuals with SCD and aMCI showed significantly reduced amplitude of low-frequency fluctuation (ALFF) values in the slow-5 band (0.01–0.027 Hz) of the right SFG and the slow-4 band (0.027–0.073 Hz) of the left SFG, respectively (Wang et al., 2021). The reduction in ALFF has been associated with deterioration in working memory (Takeuchi et al., 2017; Yoncheva et al., 2017) and episodic memory (Veldsman et al., 2017; Shi et al., 2020). Taken together, although the SCD subjects in this study had normal cognitive function as evaluated by objective tests, they demonstrated alterations of WM in the left SFG, which is highly associated with memory function according to the existing literature. Our data also suggest that the inter-voxel WM integrity of left SFG can be used to monitor the changes in memory function as the disease progresses.

In addition to the posterior cingulate cortex and precuneus, ACC is one of the main regions of the default-mode network implicated in the AD spectrum. Previous resting-state functional MRI studies have exhibited reduced functional connectivity of ACC in patients with MCI/AD compared with the HC group (Cao et al., 2020; Ibrahim et al., 2021). A recent study, which used sample entropy (SE) in measuring the complexity of dynamic BOLD signal, has further indicated that individuals with lower SE were associated with a 3.4-fold increased risk of progression to AD in comparison with those with high SE (Zheng et al., 2020). Our results further showed reduced DA of right ACC in the SCD vs. HC groups. DA reflects the magnitudes of diffusion parallel to WM tracts, and a lower DA value might be associated with axonal injury (Budde et al., 2009). Although the majority of MRI studies did not find significant GM atrophy of ACC in the SCD group compared with the HC group, our data provided evidence of microstructural changes of right ACC in this preclinical population. However, there existed some controversial results, showing that those with SCD demonstrated increased DA values of ACC compared with the HC group (Li et al., 2016). The heterogeneity of the SCD subjects might be one of the major reasons leading to the inconsistency. In our samples, there were no significant differences in most neuropsychological tests between the HC and SCD groups, while the SCD subjects in the study by Li et al. (2016) exhibited significant reduced performance of immediate recall and delayed recall. Taken together, it merits future research with a larger sample size and a less heterogeneity of subject characteristics to depict the exact profile of DA parameter in the comparisons of the HC and SCD groups.

Although previous studies have shown that alterations of WM indicators in individuals with HC/SCD were correlated with the performance of episodic memory function (Wang et al., 2012; Li et al., 2016; Luo et al., 2019; Wen et al., 2019) and general cognition (Wang et al., 2012; Luo et al., 2019), no significant associations between WM metrics and cognitive function were found in this study. A tempting interpretation of the controversial results was due to different participant characteristics. It is worth noting that individuals with SCD from most of the previous studies showed a significant decline in cognitive function as compared with the HC group (Wang et al., 2012; Hong et al., 2015; Li et al., 2016; Shao et al., 2019). Therefore, the pooling of the HC and SCD groups in the scatter plot would be easier to detect a significant association between DTI metrics and scores of cognitive tests. However, in this study, we adopted more stringent criteria to select SCD subjects who performed as well as the HC in the cognitive tests. Since the performance of neuropsychological assessments was relatively intact in the SCD group compared with the HC group, it might be difficult to find a significant correlation even pooling all the HC and SCD subjects together.

Surprisingly, individuals with SCD demonstrated higher LDH values in the left LG compared with the HC group. A previous resting-state functional MRI study also showed that compared with the HC group, the SCD group exhibited increased ALFF values of the slow-4 band in the right LG (Wang et al., 2021), suggesting a compensatory mechanism for episodic memory decline (Di et al., 2013; Yang et al., 2018). In this study, we explicitly tested this compensation hypothesis by correlating the LDH of the left LG with other significant DTI metrics. Our results indicated that higher values of LDH in the left LG were associated with lower values of LDH in the left SFG (Figure 4). This finding suggests that better inter-voxel integrity of WM (i.e., higher LDH) in the left LG would compensate for worse inter-voxel integrity of WM (i.e., lower LDH) in the left SFG, further leading to relatively intact cognitive performance. The compensation account has been evident from previous neuroimaging studies using functional MRI or magnetoencephalography (Mattay et al., 2006; Carp et al., 2010; Reuter-Lorenz and Park, 2010; Lin et al., 2018). Our current result extended prior knowledge to support the compensation hypothesis at the level of WM structure.

Several limitations should be considered when interpreting the present results. First, the sample size was relatively small, which may have decreased statistical power in the comparisons of DTI parameters between the HC and SCD groups. Second, the resolution of DTI acquisition (i.e., 256 × 256) and the number of diffusion directions (i.e., 30) are small, which might lead to less accurate results. Furthermore, although this study focused on the analysis of DTI metrics, it merits future research to investigate changes in gray matter and WM structures through voxel-based morphometry or deformation-based statistics. Third, one might argue that individuals with SCD represent a heterogeneous population, and psycho-affective factors such as subclinical depression might also contribute to the symptoms of SCD. To avoid this confounding factor, subjects who scored > 5 in the GDS were excluded from this study (Yesavage et al., 1982; Buckley et al., 2016; Sun et al., 2021). Furthermore, it should be noted that our SCD subjects were proactive help-seekers for their worries about cognitive decline, which has been linked to an increased risk in the development of AD/aMCI (Miebach et al., 2019). Fourth, this was a cross-sectional study and thus was insufficient to predict the clinical outcome of SCD. The pattern of longitudinal changes in WM microstructures requires further confirmation by larger-sample, follow-up studies.

## Conclusion

This is the first study to report that individuals with SCD show alterations of inter-voxel WM integrity in the left SFG and LG, as indexed by LDH parameters, compared with those without SCD. Reduced DA of right ACC was also observed in the SCD group vs. the control group. Our data suggest that individuals with SCD might present detectable changes in WM structures before they demonstrate objective cognitive dysfunction.

## Data Availability Statement

The original contributions presented in the study are included in the article/supplementary material, further inquiries can be directed to the corresponding author.

## Ethics Statement

The studies involving human participants were reviewed and approved by the Institutional Review Board of Taipei Veterans General Hospital. The patients/participants provided their written informed consent to participate in this study.

## Author Contributions

Y-PC, P-NW, and C-HC: include conception and study design. P-NW and C-HC: data collection and acquisition. Y-PC and P-TBL: statistical analysis. Y-PC, P-TBL, and C-HC: drafting the manuscript work or revising it critically for important intellectual content. All authors interpretation of results, approval of the final version to be published, and agreement to be accountable for the integrity and accuracy of all aspects of the work.

## Conflict of Interest

The authors declare that the research was conducted in the absence of any commercial or financial relationships that could be construed as a potential conflict of interest.

## Publisher’s Note

All claims expressed in this article are solely those of the authors and do not necessarily represent those of their affiliated organizations, or those of the publisher, the editors and the reviewers. Any product that may be evaluated in this article, or claim that may be made by its manufacturer, is not guaranteed or endorsed by the publisher.

## Footnotes

1
http://www.fmrib.ox.ac.uk/fsl

2
https://fsl.fmrib.ox.ac.uk/fsl/fslwiki/TBSS

3
http://dbm.neuro.uni-jena.de/tfce/
